# Comparative Proteomic Analysis of Mature and Immature Oocytes of the Swamp Buffalo (*Bubalus bubalis*)

**DOI:** 10.3390/ijms17010094

**Published:** 2016-01-13

**Authors:** Qiang Fu, Zhen-Fang Liu, Yu-Lin Huang, Yang-Qing Lu, Ming Zhang

**Affiliations:** 1 State Key Laboratory of Subtropical Agro-Bioresource Conservation and Utilization, Guangxi University, Nanning 530004, Guangxi, China; gxfuq@163.com (Q.F.); echomide@163.com (Y.-L.H.); luyangqing@126.com (Y.-Q.L.); 2 Reproductive Medicine Center, Xiamen 361000, Fujian, China; liuzhenfang@126.com

**Keywords:** swamp buffalo, oocyte, comparative proteomics, heat shock protein 60 (HSP60), Gem-associated Protein 8 (GEMIN8), Western blotting

## Abstract

Maternal protein components change markedly during mammalian oogenesis. Many of these proteins have yet to be characterized and verified. In this study, a proteomics approach was used to evaluate changes in proteins during oogenesis in the Swamp Buffalo (*Bubalus bubalis*). Proteins from 500 immature oocytes and 500 *in vitro* matured oocytes were subjected to two-dimensional electrophoresis, and more than 400 spots were detected. Image analysis indicated that 17 proteins were differentially expressed between the two groups. Eight proteins were identified by mass spectrometry. In mature oocytes, three proteins were down-regulated: major vault protein (MVP), *N*-acetyllactosaminide β-1,6-*N*-acetylglucosaminyl-transferase (GCNT-2), and gem-associated protein (GEMIN)8, whereas five other proteins, heat shock protein (HSP)60, Ras-responsive element-binding protein 1 (RREB-1), heat shock cognate 71 kDa protein (HSC71), hemoglobin subunit α (HBA), and BMP-2-inducible protein kinase (BMP-2K), were up-regulated. The expression profiles of HSP60 and GEMIN8 were further verified by Western blotting. The changes in HSP60 protein expression demonstrate the increasing need for mitochondrial protein importation to facilitate macromolecular assembly during oocyte maturation. The down-regulation of GEMIN8 production implies that RNA splicing is impaired in mature oocytes.

## 1. Introduction

Mammalian oocytes undergo a series of regulated events during follicular development in the ovary, from primordial follicles to ovulation. An oocyte reaches its full size at the germinal vesicle (GV) stage. Here, mRNA synthesis ceases and the nucleus remains intact [1]. The oocytes then resume meiosis in response to specific signals. GV breakdown (GVBD) occurs, which allows exposure of the nuclear material to the cytoplasm. Following GVBD, the chromatin condenses and the first polar body is extruded from the oocytes. Subsequently, oocytes mature to the metaphase II (MII) stage of meiosis and undergo ovulation [2]. During the developmental oocyte maturation, many of the changes are mediated by dynamic variations in protein composition and by post-translational modifications in cumulus-oocyte complexes (COCs). Some well-known oocyte proteins, such as Stella [3], Zar1 [4], and Mater [5], play important roles in zygote activation. Many aspects of these mechanisms have been studied in order to understand the molecular foundations of oocyte maturation, and proteomic technology has been used for some species. Calvert *et al.* [6] carried out a pioneering study; they identified eight highly abundant heat shock proteins (HSPs) and related chaperones in the mature, MII-arrested mouse oocyte (zonae pellucidae were removed) using two-dimensional electrophoresis (2-DE). Vitale *et al.* (2007) [1] identified 12 proteins that appeared to be differentially expressed between GV- and MII-stage murine oocytes using 2-DE combined with mass spectrometry (MS). The proteomes of matured mouse COCs were constructed, and 259 protein spots, corresponding to 156 individual proteins, have been identified [7]. Ellederova *et al.* (2004) [8] first found that antiquitin was increased significantly in MI and MII, compared with GV stages in pig oocytes. A large-scale protein identification strategy was also performed for oocyte proteomic analysis: 4395 proteins were expressed in bovine COCs; 1092 were expressed in oocytes; and 858 were common to both COCs and oocytes [9]. Zhang *et al.* (2009) [10] identified a total of 625 different proteins from 2700 mature oocytes, lacking zona pellucidae, using SDS–PAGE combined with high performance liquid chromatography (HPLC). They screened 76 maternal proteins with high levels of mRNA expression, both in oocytes and zygotes. Recently, 2781 and 2973 proteins were successfully identified from GV stage and MII stage oocytes, respectively. The proteome of oocytes provides us with important information on the factors regulating the developmental competence of oocytes [11]. Pfeiffer *et al.* [12] present the proteome of MII mouse oocytes to a depth of 3699 proteins, which substantially extends the number of proteins identified until now in mouse oocytes. Efforts to search for reprogramming factors in embryonic stem cells has been conducted by Graumann *et al.* [13]. More than 5000 distinct proteins were quantified including some important stem cell reprogramming markers.

Although most basic reproductive biology studies have been done in the mouse and other species, no proteomic study has been reported to date for buffalo oocytes. Swamp or water buffalos (*Bubalus bubalis*) are adapted to hot–humid tropical climatic conditions, but have low reproductive efficiency. Basic research on oocyte developmental biology should contribute to improving buffalo fertility, and will also be important for improving genetic traits and the efficacy of *in vitro* maturation (IVM) systems. During the procedure of meiotic maturation, oocytes in GV stage undergo a variety of events: chromosomal rearrangement, formation of spindle and polar body, and movement of cortical granules. Here, we compared proteins from immature and mature buffalo oocytes using a 2DE–MS strategy. Our objective was to develop a 2DE method for discovering proteins that are differentially expressed between immature and mature oocytes. Furthermore, we identified and verified some target proteins that might be involved in oocyte maturation in swamp buffalo.

## 2. Results

### 2.1. Two-Dimensional Gel Electrophoresis Profile

**Figure 1:**
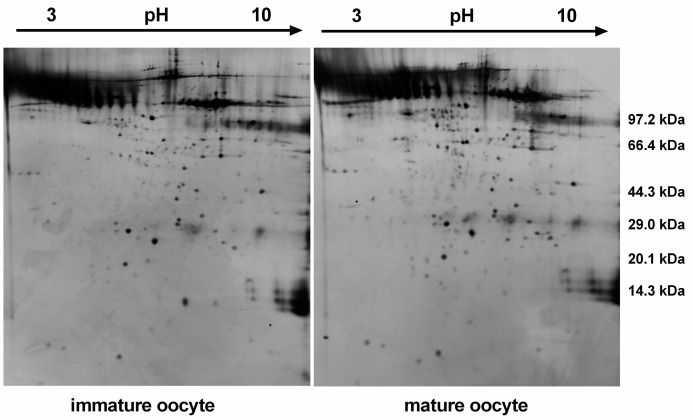
Images of silver nitrate-stained 2-DE gels from immature and mature oocytes. Proteins were separated on a pH 3–10 gradient using nonlinear (NL) immobilized pH Gradient (IPG) strips in the first dimension and 12.5% SDS–PAGE in the second dimension.

**Figure 2:**
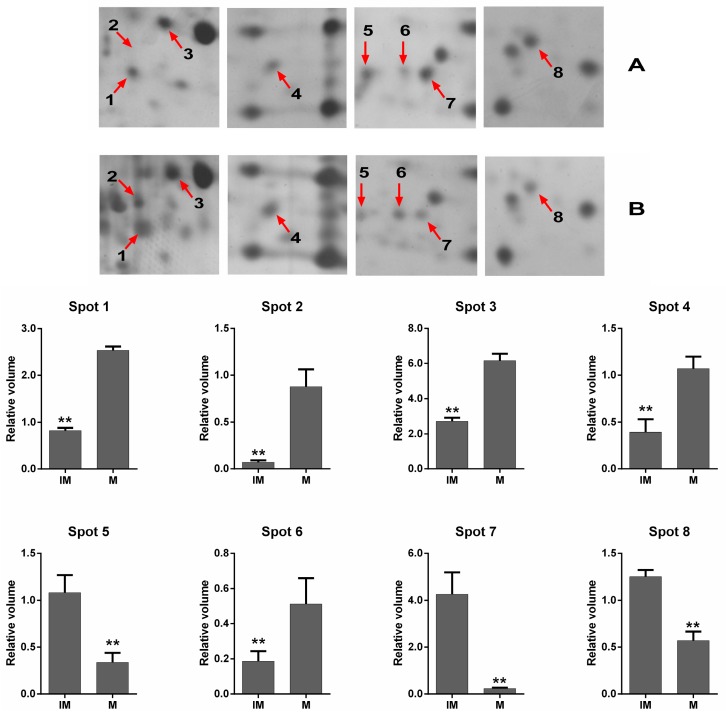
Magnified maps of differentially expressed protein spots and statistical analysis. (**A**) The magnified 2-DE maps from immature oocytes; (**B**) the magnified 2-DE maps from mature oocytes.IM: immature oocyte; M: mature oocyte. Spot 1: IM (0.82 ± 0.06) *vs.* M (2.54 ± 0.08); Spot 2: IM (0.07 ± 0.02) *vs.* M (0.87 ± 0.16); Spot 3: IM (2.71 ± 0.20) *vs.* M (6.16 ± 0.41); Spot 4: IM (0.39 ± 0.14) *vs.* M (1.07 ± 0.13); Spot 5: IM (1.08 ± 0.19) *vs.* M (0.34 ± 0.10); Spot 6: IM (0.19 ± 0.06) *vs.* M (0.51 ± 0.15); Spot 7: IM (4.26 ± 0.93) *vs.* M (0.24 ± 0.04); Spot 8: IM (1.25 ± 0.07) *vs.* M (0.57 ± 0.10). ** Denotes significantly different (*p* < 0.01).

Proteins were extracted from a total of 500 immature and 500 mature oocytes, separated on 2DE gels in a nonlinear gradient (pH 3–10), and then subjected to SDS–PAGE. Silver-stained gels are shown in Figure 1. On average, approximately 400 protein spots were distributed in a molecular weight (*M*_W_) range between ~20 and ~150 kDa. Most protein spots shared a similar *M*_W_ and pI. The 2-DE image of immature oocytes was set as the control. ImageMaster 5.0 software analyses showed that 17 protein spots were significantly different in OD (at least a 2.5-fold change). Six protein spots were downregulated in the immature oocyte group, while 11 protein spots were upregulated in the mature oocyte group. All 17 differentially expressed protein spots were excised from their respective gels, then digested in-gel with trypsin, and subsequently analyzed by Matrix-Assisted Laser Desorption Ionization Time of Flight (MALDI-TOF/TOF) mass spectrometry. Out of them, 47% (8/17) of the excised spots were identified successfully with a Mascot score >60 (confidence interval, CI > 95%). The eight identified proteins and their variations in expression are shown on magnified 2-DE gel maps (Figure 2). Five protein spots were increased significantly in the mature oocyte group (Spots 1, 2, 3, 4, and 6), while three spots were significantly decreased (Spots 5, 7, and 8).

### 2.2. MS Identification and Bioinformatics Analysis

The raw mass spectra data were searched against the Bostaurus proteome database [14] using the Mascot algorithm. The results are listed in Table 1. Expert Protein Analysis System (ExPASy) [15] was used for a preliminary functional analysis of these proteins. The MVP, HSC71, and GEMIN8 proteins are located in the cytoplasm and nucleus, whereas the RREB1 and BMP2K proteins are located in the nucleus. The HSP60 protein is located in mitochondria. The HSC71 protein is involved in estrogen signaling and spliceosome pathways.

**Table 1:** The identification of proteins by MALDI-TOF/TOF mass spectrometry.

Spot No.	Protein Name	Gene Symbol	Calc.MW (Da)	Uniprot Accession No.	Peptide Count	Mascot Score *	Regulation Profile	Subcellular Location
1	Ras-responsive element-binding protein 1	RREB1	181,420	Q92766	1	76 (99%)	mature up	Nucleus
2	Hemoglobin subunit α	HBA	15,184	P01966	1	85 (99%)	mature up	Unknown
3	Heat shock cognate 71 kDa protein	HSC71	71,241	P19120	2	66 (99%)	mature up	Cytoplasm/Nucleus
4	60kDa heat shock protein, mitochondrial	HSP60	61,108	P31081	3	117 (100%)	mature up	Mitochondrion
5	*N*-acetyllactosaminide β-1,6-*N*-acetylglucosaminyl-transferase, isoform C	GCNT2	46,531	Q8NFS9	5	63 (98%)	immature up	Golgi apparatus/Membrane
6	Major vault protein	MVP	98,924	Q3SYU9	11	190 (100%)	immature up	Cytoplasm/Nucleus
7	BMP-2-inducible protein kinase	BMP2K	129,172	Q9NSY1	4	83 (99%)	mature up	Nucleus
8	Gem-associated protein 8	GEMIN8	26,744	Q1LZ79	3	86 (99%)	immature up	Cytoplasm/Nucleus

* the confidence interval (C.I.) of mascot score were list in the branket.

### 2.3. Western Blot Validation

**Figure 3:**
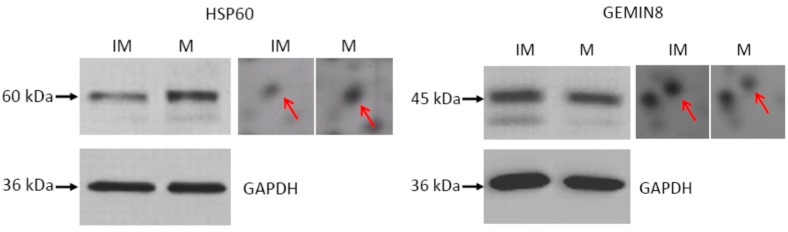
Validation of HSP60 and GEMIN8 proteins by Western blotting. IM, immature oocytes; M, mature oocytes. GAPDH was used as a loading control. The locations of HSP60 protein and GEMIN8 protein on 2-DE images were denoted by the red arrows.

Western blotting was performed to confirm the expressions of HSP60 and GEMIN8 proteins. As shown in Figure 3, GAPDH protein was used as a loading control. For the HSP60 protein, there was a weaker band in the gel for immature oocytes compared with that for mature oocytes. The Western blot results were consistent with those of the 2-DE gels, indicating that the results of these analyses were reliable. The Western blot signals of the two GEMIN8 protein bands were similar between mature and immature oocytes. Using optical density analysis, the expression profile exhibited significant differences between immature and mature oocytes.

## 3. Discussion

The protein content of oocytes is very important for fertilization and pregnancy outcomes. The oocyte proteome is a direct representation of the factors that decide its phenotype and its developmental potential. Here, we used comparative proteomics to perform a preliminary analysis of proteins that were differentially expressed between immature and mature buffalo oocytes. Only 400 proteins were detected in the 2-DE gel images, which account for 1.7% of bovine proteome database (24,113 sequences). The lower coverage attributed to the disadvantage of 2-DE. Certain proteins are difficult to separate by 2-DE, including those that are in low abundance, hydrophobic, acidic, basic, very large, or very small proteins [16]. We believe that more proteins will be identified after zona pellucida (ZP) is removed, since the ZP1, ZP2, and ZP3 proteins are highly abundant. As shown in Figure 1, it was evident that the presence of abundant ZP proteins blocked out many of the protein spots. It is possible to use ZP-free oocytes for isolating oocyte-specific proteins using 2-DE [10,11]. On the other hand, 2-DE-MS and LC-MS are known to be two complementary strategies. Using 2-DE is a classic method to display changes in protein spot visually, while high-throughput LC-MS strategy can identify total protein or give a quantitative analysis [17,18].

In this study, six proteins were decreased in mature oocytes, while 11 proteins were increased. This increase in upregulation is consistent with the view that MII oocytes have higher translational activity than immature ones [19]. All seventeen protein spots were excised and prepared for MALDI-TOF analysis. Eight were successfully identified. Attempts to identify the other nine failed because of their low abundance. Silver-staining has a higher sensitivity than Commassie blue staining. Protein spots with low OD density on silver-stained gels were not necessary for successful identification of MS. In addition, buffalo genomic database annotation has not been accomplished and the proteome database has not yet been released. Incomplete knowledge of the buffalo proteome may have restricted the ratio of protein identification.

The HSP60 and GEMIN8 proteins were selected for verification using Western blotting. As shown in Figure 3, the similar bands for GAPDH between runs demonstrated that the Western blot results were reliable. The optical density of the HSP60 band indicated that the Western blot signals were consistent with those of the 2-DE results. Change in the expression of the HSPs protein family implies the increasing need for translated proteins and response to heat stress [20]. Calvert *et al.* [6] identified several HSPs (HSP70 and HSP90) and related chaperones in mature mouse egg using 2-DE. HSP70 is one of the first genes for zygotic gene activation and is constitutively synthesized in the immature, preovulatory mouse oocyte [21]. Levels of HSP70 mRNA decrease following oocyte maturation and ovulation [22]. The HSP60 protein is located in the mitochondrial matrix, where it plays a role in macromolecular assembly and might facilitate the correct folding of imported proteins [23]. The regulation of HSP60 during mouse embryonic stem cell differentiation has already been reported [24,25,26]. The biological role of HSP60 in buffalo or other mammalian oocyte maturation is not yet known. In this study, HSP60 protein was found to be upregulated in mature oocytes. We inferred that variation of HSP60 expression levels was related to the increase of mitochondria in mature oocytes. The function of HSP60 during oocyte maturation needs to be investigated in further research. The relative amount of HSC71 protein also showed a trend for upregulation in mature oocytes. This protein acts as one of the molecular chaperones, which assists proteins in folding from their denatured state to the correctly folded product [27]. Previous studies suggested that both constitutive and inducible forms of HSC71 and the HSP protein family are translated during bovine oocyte maturation and early embryo development, and HSC71 is also translated during pig oocyte maturation [20,28].

The GEMIN8 protein was also subjected to Western blotting for verification. RNA interference experiments indicated that GEMIN8 gene knockdown impairs snRNP assembly [29]. Gemin8 cDNA encodes for a protein of 234 amino acids with a predicted molecular mass of 28 kDa. In the present study, the theoretical isoelectric point (pI 6.8) coincided with the location in 2-DE images. However, the protein spot of Gemin8 was located in 44 kDa. Western blot results indicated two hybrid bands with a range of 35–45 kDa. One possible reason is that many other homologous proteins disturbed the Western blot assay. The GEMIN protein family is involved in the formation of survival motor neuron (SMN) complexes involved in small nuclear ribonucleic protein (snRNP) assembly [30]. Gemin8 is a novel integral component of cellular SMN complexes. Gemin8 binds directly and efficiently to the Gemin6–Gemin7 complex [29].

The BMP2K protein encodes a serine/threonine kinase protein and might affect the transcriptional activities of target genes. Recently, BMP2K was identified as a clathrin-coated vesicle-associated protein, suggesting that it might also function to regulate endocytic complexes [31]. The MVP protein, also called lung resistance-related protein, is a ribonucleoprotein, comprising a major part (>70%) of vault large ribonucleoprotein particles [32]. There appears to be a single, evolutionarily-conserved MVP gene in humans and other mammals [33]. The function of vault particles is not known; they might act as multi-subunit structures, and serve as scaffolds for proteins involved in signal transduction. The MVP protein showed a predominantly cytoplasmic distribution in normal porcine oocytes and embryos at the GV, M-II, 1-cell, 2-cell, 8- to 16-cell, morula and blastocyst stages of preimplantation development, *in vitro* and *in vivo* [34]. In our study, the MVP protein accumulated in immature oocytes, but was decreased in mature oocytes. Another protein, hemoglobin, was not supposed to be present in oocytes; however, this protein was identified in buffalo oocytes, not only in 2-DE experiments, but also in the shotgun proteomics approach based on LC-MS/MS. We suspected that the high expression of the hemoglobin protein in mature oocytes may be attributed to being contaminated by the addition of fetal bovine serum into the culture medium.

## 4. Materials and Methods

### 4.1. Oocyte in Vitro Maturation

Oocyte collection and maturation were done as described [35]. Briefly, immature oocytes were aspirated with a syringe from follicles 2–6 mm in diameter, from abattoir-derived ovaries. Immature oocytes were washed in TCM199 (Gibco, Grand Island, NY, USA) supplemented with 0.3% bovine serum albumin and once more in maturation medium: TCM199 supplemented with 5% estrous bovine serum and 10 µg/mL FSH. They were cultured in sterile mineral oil at 38.5 °C under 5% CO_2_ in humidified air for 22–24 h. Following maturation, the oocytes with expanding cumulus cells were selected for proteomic analysis. The cumulus cells were stripped off by repeated pipetting.

### 4.2. Sample Perparation and Protein Extraction

Pooled groups of 500 immature and 500 mature oocytes were lysed in 30 µL of lysis buffer (7 M urea, 2 M thiourea, 4% *w*/*v* CHAPS zwitterionic detergent, 0.5% dithiothreitol (DTT), and 1 mM phenylmethanesulfonyl fluoride (PMSF) proteinase inhibitor, pH 8.0) for 30 min at room temperature. Samples were homogenized by repeated pipetting. After being centrifuged (4 °C, 10 min, 12,000× *g*), the supernatant was transferred to a new 1.5 mL tube for further processing. After being precipitated with ice-cold acetone, protein concentrations were determined using a Bradford protein assay. The samples were stored at −80 °C for subsequent thawing and 2-DE.

### 4.3. Two-Dimensional Electrophoresis

The protein samples were solubilized in rehydration buffer (8 M urea, 2% CHAPS, 15 mM DTT, and 0.5% immobilized pH gradient (IPG) buffer pH range 3–10). For isoelectric focusing (IEF), denatured proteins were separated using 13 cm IPG Strips (pH 3–10) with an IPGphor III IEF system (GE Healthcare Life Sciences, Marlborough, MA, USA). The IEF was done in five steps: active rehydration at 30 V for 6 h; 60 V for 6 h; 500 V for 1 h; 1000 V for 1 h; then increased to 5000 V for 1 h, and increased gradually until a total of 50,000 V/h was reached. After IEF separation, the gel strips were equilibrated twice for 15 min. The first equilibration buffer contained 50 mM Tris-HCl (pH 8.8), 8 M urea, 30% (*v*/*v*) glycerol, and 2% SDS with 1% (*w*/*v*) DTT. The second equilibration buffer contained the same ingredients except that DTT was replaced with 2.5% (*w*/*v*) iodoacetamide (IAA).

After equilibration, the IPG strips were rinsed to remove excess equilibration buffer, and applied to a vertical linear gradient SDS–PAGE (12%, 17 × 17 cm, 1 mm thick). SDS–PAGE was carried out using an EttanDALTsix electrophoresis system (GE Healthcare Life Sciences), according to the supplier’s instructions.

### 4.4. Silver Staining and Image Analysis

The 2-DE gels were silver-stained using a MS-compatible method without formaldehyde. Gels were scanned in transparency mode and the images were analyzed using ImageMaster 2D software (GE Healthcare Life Sciences). The protein spots were considered to be up- or downregulated if their optical density (OD) measures showed an increase or a decrease by at least a factor of 2.5, respectively.

### 4.5. In-Gel Digestion and Mass Spectrometry Identification

After differential analysis of the gels, protein spots were excised for digestion. Briefly, gel fragments were discolored in a solution containing 30 mM K_3_Fe(CN)_6_ and 100 mM Na_2_S_2_O_3_, then washed twice in 40 mM NH_4_HCO_3_ solution and dehydrated with 100 µL of acetonitrile, and incubated overnight at 37 °C with 10 ng/µL trypsin. Digested peptides were extracted with 50% acetonitrile and dried, then resuspended in 1 µL of Milli-Q water with 0.1% trifluoroacetic acid (TFA) and spotted onto a MALDI target. α-Cyano-4-hydroxycinnamic acid (CHCA) solutionwas used as a covering matrix. The peptides were run through a MALDI-TOF/TOF mass spectrometry analyzer (4800 Plus, ABSciex Inc., Foster City, CA, USA). Acquired MS spectra ranged from 800–4000 Da in reflector-positive mode. For each spot scan, 10 of the most intense precursor ions were selected for tandem MS/MS analysis.

### 4.6. Protein Bioinformation Analysis

All the differentially expressed proteins were analyzed to determine their function using the ExPASy website resource [36]. Gene ontogeny (GO) analyses were performed using KOBAS 2.0 [37].

### 4.7. Western Blot Validation

Each of the 20 µg protein samples of immature and mature oocytes were separated by SDS–PAGE (12%, 13 cm × 13 cm). After electrophoresis, the protein lanes were transferred to polyvinylidene fluoride (PVDF) membranes (Millipore Corp., Bedford, MA, USA) using an electronic transfer apparatus (Bio-Rad Laboratories, Inc., Hercules, CA, USA) at 70 V for 2 h. Nonspecific binding sites on PVDF membranes were blocked by incubating them for 1 h at room temperature with 5% skim milk in phosphate-buffered saline (PBS) with 0.1% Tween-20. Membranes were incubated overnight at 4 °C with rabbit polyclonal antibodies against Heat Shock Protein (HSP)60 and Gem-associated Protein (GEMIN)8 (diluted 1:1000, CWbio Co., Ltd., Beijing, China). They were washed with PBS plus 0.1% Tween-20 three times, and incubated with a secondary antibody (diluted 1:3000, CWbio Co.) for 1 h at room temperature. Finally, the membranes were washed and visualized using autoradiography (X-ray) film. Glyceraldehyde 3-phosphate dehydrogenase (GAPDH) was used as an internal loading control.

## 5. Conclusions

Eight proteins were expressed differentially between immature and mature oocytes. The changes in HSP60 protein expression demonstrate the increasing need for mitochondrial protein importation and macromolecular assembly during oocyte maturation. Downregulation of the GEMIN8 protein implies that RNA splicing is impaired in mature oocytes. These differentially expressed proteins could be used as biomarkers during *in vitro* maturation and will provide new information for understanding the mechanism of buffalo oogenesis.

## References

[1] Vitale A.M., Calvert M.E., Mallavarapu M., Yurttas P., Perlin J., Herr J., Coonrod S. (2007). Proteomic profiling of murine oocyte maturation. Mol. Reprod. Dev..

[2] Motlik J., Fulka J. (1981). *In vitro* maturation of mammalian oocytes. Ontogenez.

[3] Payer B., Saitou M., Barton S.C., Thresher R., Dixon J.P., Zahn D., Colledge W.H., Carlton M.B., Nakano T., Surani M.A. (2003). Stella is a maternal effect gene required for normal early development in mice. Curr. Biol..

[4] Wu X., Viveiros M.M., Eppig J.J., Bai Y., Fitzpatrick S.L., Matzuk M.M. (2003). Zygote arrest 1 (*Zar1*) is a novel maternal-effect gene critical for the oocyte-to-embryo transition. Nat. Genet..

[5] Tong Z.B., Gold L., Pfeifer K.E., Dorward H., Lee E., Bondy C.A., Dean J., Nelson L.M. (2000). Mater, a maternal effect gene required for early embryonic development in mice. Nat. Genet..

[6] Calvert M.E., Digilio L.C., Herr J.C., Coonrod S.A. (2003). Oolemmal proteomics—Identification of highly abundant heat shock proteins and molecular chaperones in the mature mouse egg and their localization on the plasma membrane. Reprod. Biol. Endocrinol..

[7] Meng Y., Liu X.H., Ma X., Shen Y., Fan L., Leng J., Liu J.Y., Sha J.H. (2007). The protein profile of mouse mature cumulus-oocyte complex. Biochim. Biophys. Acta.

[8] Ellederova Z., Halada P., Man P., Kubelka M., Motlik J., Kovarova H. (2004). Protein patterns of pig oocytes during *in vitro* maturation. Biol. Reprod..

[9] Memili E., Peddinti D., Shack L.A., Nanduri B., McCarthy F., Sagirkaya H., Burgess S.C. (2007). Bovine germinal vesicle oocyte and cumulus cell proteomics. Reproduction.

[10] Zhang P., Ni X., Guo Y., Guo X., Wang Y., Zhou Z., Huo R., Sha J. (2009). Proteomic-based identification of maternal proteins in mature mouse oocytes. BMC Genom..

[11] Wang S., Kou Z., Jing Z., Zhang Y., Guo X., Dong M., Wilmut I., Gao S. (2010). Proteome of mouse oocytes at different developmental stages. Proc. Natl. Acad. Sci. USA.

[12] Pfeiffer M.J., Siatkowski M., Paudel Y., Balbach S.T., Baeumer N., Crosetto N., Drexler H.C., Fuellen G., Boiani M. (2011). Proteomic analysis of mouse oocytes reveals 28 candidate factors of the “reprogrammome”. J. Proteome Res..

[13] Graumann J., Hubner N.C., Kim J.B., Ko K., Moser M., Kumar C., Cox J., Scholer H., Mann M. (2008). Stable isotope labeling by amino acids in cell culture (SILAC) and proteome quantitation of mouse embryonic stem cells to a depth of 5111 proteins. Mol. Cell. Proteom..

[14] Bovine proteome database. http://www.uniprot.org/proteomes/up000009136.

[15] SIB Swiss Institute of Bioinformatics Members (2015). The SIB Swiss Institute of Bioinformatics’ resources: Focus on curated databases. Nucleic Acids Res..

[16] Rocken C., Ebert M.P., Roessner A. (2004). Proteomics in pathology, research and practice. Pathol. Res. Pract..

[17] Charro N., Hood B.L., Faria D., Pacheco P., Azevedo P., Lopes C., de Almeida A.B., Couto F.M., Conrads T.P., Penque D. (2011). Serum proteomics signature of cystic fibrosis patients: a complementary 2-DE and LC-MS/MS approach. J. Proteom..

[18] Hammer E., Bien S., Salazar M.G., Steil L., Scharf C., Hildebrandt P., Schroeder H.W., Kroemer H.K., Volker U., Ritter C.A. (2010). Proteomic analysis of doxorubicin-induced changes in the proteome of HepG2cells combining 2-D DIGE and LC-MS/MS approaches. Proteomics.

[19] Oh B., Hwang S.Y., Solter D., Knowles B.B. (1997). Spindlin, a major maternal transcript expressed in the mouse during the transition from oocyte to embryo. Development.

[20] Novak S., Paradis F., Savard C., Tremblay K., Sirard M.A. (2004). Identification of porcine oocyte proteins that are associated with somatic cell nuclei after co-incubation. Biol. Reprod..

[21] Curci A., Bevilacqua A., Mangia F. (1987). Lack of heat-shock response in preovulatory mouse oocytes. Dev. Biol..

[22] Manejwala F.M., Logan C.Y., Schultz R.M. (1991). Regulation of hsp70 mRNA levels during oocyte maturation and zygotic gene activation in the mouse. Dev. Biol..

[23] Arya R., Mallik M., Lakhotia S.C. (2007). Heat shock genes—Integrating cell survival and death. J. Biosci..

[24] Wang D., Gao L. (2005). Proteomic analysis of neural differentiation of mouse embryonic stem cells. Proteomics.

[25] Kurisaki A., Hamazaki T.S., Okabayashi K., Iida T., Nishine T., Chonan R., Kido H., Tsunasawa S., Nishimura O., Asashima M. (2005). Chromatin-related proteins in pluripotent mouse embryonic stem cells are downregulated after removal of leukemia inhibitory factor. Biochem. Biophys. Res. Commun..

[26] Intawicha P., Wang S.H., Hsieh Y.C., Lo N.W., Lee K.H., Huang S.Y., Ju J.C. (2013). Proteomic profiling of rabbit embryonic stem cells derived from parthenotes and fertilized embryos. PLoS ONE.

[27] Bukau B., Weissman J., Horwich A. (2006). Molecular chaperones and protein quality control. Cell.

[28] Edwards J.L., Hansen P.J. (1997). Differential responses of bovine oocytes and preimplantation embryos to heat shock. Mol. Reprod. Dev..

[29] Carissimi C., Saieva L., Baccon J., Chiarella P., Maiolica A., Sawyer A., Rappsilber J., Pellizzoni L. (2006). Gemin8 is a novel component of the survival motor neuron complex and functions in small nuclear ribonucleoprotein assembly. J. Biol. Chem..

[30] Carissimi C., Saieva L., Gabanella F., Pellizzoni L. (2006). Gemin8 is required for the architecture and function of the survival motor neuron complex. J. Biol. Chem..

[31] Borner G.H., Antrobus R., Hirst J., Bhumbra G.S., Kozik P., Jackson L.P., Sahlender D.A., Robinson M.S. (2012). Multivariate proteomic profiling identifies novel accessory proteins of coated vesicles. J. Cell Biol..

[32] Kedersha N.L., Heuser J.E., Chugani D.C., Rome L.H. (1991). Vaults. III. Vault ribonucleoprotein particles open into flower-like structures with octagonal symmetry. J. Cell Biol..

[33] Mossink M., van Zon A., Franzel-Luiten E., Schoester M., Scheffer G.L., Scheper R.J., Sonneveld P., Wiemer E.A. (2002). The genomic sequence of the murine major vault protein and its promoter. Gene.

[34] Sutovsky P., Manandhar G., Laurincik J., Letko J., Caamano J.N., Day B.N., Lai L., Prather R.S., Sharpe-Timms K.L., Zimmer R. (2005). Expression and proteasomal degradation of the major vault protein (MVP) in mammalian oocytes and zygotes. Reproduction.

[35] Zhang M., Lu K.H., Seidel G.E. (2003). Development of bovine embryos after *in vitro* fertilization of oocytes with flow cytometrically sorted, stained and unsorted sperm from different bulls. Theriogenology.

[36] Expert Protein Analysis System (ExPASy). http://www.expasy.org.

[37] KEGG Orthology Based Annotation System (KOBAS). http://kobas.cbi.pku.edu.cn.

